# Characteristics and prognosis of patients with cirrhosis presenting with acute respiratory distress syndrome: A bicentric retrospective study

**DOI:** 10.1016/j.jointm.2025.12.008

**Published:** 2026-02-20

**Authors:** Adam Celier, Marie-Amélie Ordan, Aymeric Lanore, Julien Mayaux, Philippe Ichaï, Marika Rudler, Maxens Decavèle, Alexandre Demoule

**Affiliations:** 1 AP-HP, Groupe Hospitalier Universitaire APHP-Sorbonne Université, site Pitié-Salpêtrière, Service de Médecine Intensive et Réanimation (Département R3S), Paris, France; 2 Unité de Soins Intensifs d’Hépatologie, AP-HP Hôpital Paul-Brousse, Centre Hépato-Biliaire, Villejuif, France; 3 Sorbonne Université, Institut du Cerveau - Paris Brain Institute - ICM, Inserm, CNRS, Paris, France; 4 AP-HP, Groupe Hospitalier Universitaire APHP-Sorbonne Université, site Pitié-Salpêtrière, Département de neurologie, Centre d’Investigation Clinique Neurosciences, Hôpital Pitié-Salpêtrière, Paris, France; 5 AP-HP, Groupe Hospitalier Universitaire APHP-Sorbonne Université, site Pitié-Salpêtrière, Unité de soins intensifs de gastro-entérologie, Département d’hépato-gastro-entérologie, Paris, France; 6 INSERM UMR_S 938, Centre de Recherche Saint-Antoine, Maladies Métaboliques, Biliaires et Fibro-Inflammatoire du Foie, Institute of Cardiometabolism and Nutrition (ICAN), Paris, France; 7 UMRS1158 Neurophysiologie Respiratoire Expérimentale et Clinique, Sorbonne Université, INSERM, Paris, France

**Keywords:** Cirrhosis, Acute respiratory distress syndrome, Acute on chronic liver failure

## Abstract

**Background:**

Data on acute respiratory distress syndrome (ARDS) are scarce in patients with cirrhosis admitted to the intensive care unit (ICU). This study aimed to assess 28-day mortality among ventilated patients with cirrhosis presenting with ARDS and identify associated factors.

**Methods:**

We conducted a retrospective observational cohort study of consecutive mechanically ventilated patients with cirrhosis admitted to two ICUs between 1 February 2007 and 31 December 2021 and fulfilling ARDS criteria during ICU stay. Demographic, clinical, and biological data were collected, and factors associated with 28-day mortality were identified using multivariable logistic regression.

**Results:**

Among 621 patients with cirrhosis requiring mechanical ventilation, 165 (26.6%) met ARDS criteria during their stay. Reasons for ICU admission included acute respiratory failure (52.1%), gastrointestinal bleeding with shock (18.2%), and septic shock (15.8%). The median Model for End-Stage Liver Disease score on admission was 29 (interquartile range [IQR]: 23–36) and Simplified Acute Physiologic Score II was 60 (IQR: 44–72). ARDS developed a median of 2 (IQR: 1–5) days after ICU admission, with a partial pressure of arterial oxygen to fraction of inspired oxygen (PaO_2_/FiO_2_) ratio of 118 mmHg. Twenty-eight-day mortality was 75.2% and did not change over the study period (80.9% in 2007–2014 *vs*. 68.4% in 2015–2021, *P*=0.064). On multivariate analysis, factors associated with 28-day mortality were MELD score (odds ratio [OR]=2.31, 95% confidence interval [CI]: 1.48 to 3.80; *P* <0.001), PaO_2_/FiO_2_ ratio at ARDS onset (OR=0.49, 95% CI: 0.24 to 0.97; *P*=0.039) and ICU admission for acute respiratory failure (OR=0.44, 95% CI: 0.19 to 0.97; *P*=0.047).

**Conclusions:**

ARDS is common among patients with cirrhosis admitted to the ICU. Mortality remains high and has not improved over the past decade. Identifying factors associated with mortality may help guide treatment intensity in this population.

## Introduction

Cirrhosis imposes an increasing health burden on many countries, accounting for over 1.32 million deaths worldwide in 2017.^[^^1^^]^ Patients in late-stage cirrhosis are likely admitted to the intensive care unit (ICU) for gastrointestinal-bleeding, acute renal or respiratory failure, sepsis or coma, and account for 2% to 4% of ICU admissions.^[^^2^^,^^3^^]^ Although the prognosis of patients with cirrhosis admitted to the ICU has significantly improved during the last decades,^[^[2], [3], [4], [5], [6]^]^ in-hospital mortality remains high, ranging from 16% to 45%.^[^[4], [5], [6]^]^ Prognosis is now better characterized using specific scoring systems such as the chronic liver failure-sequential organ failure assessment (CLIF-SOFA) and CLIF Consortium Acute on Chronic Liver Failure (CLIF-C ACLF) scores, which highlight the impact of extra-hepatic organ failures.^[^^7^^,^^8^^]^

Respiratory failure occurs in 10% to 50% of critically ill patients with cirrhosis, either as the primary reason for ICU admission or developing secondarily during the ICU stay.^[^^6^^]^ Mechanical ventilation is a major risk factor for death in this population.^[^[9], [10], [11], [12]^]^ Acute respiratory distress syndrome (ARDS) is the most severe form of acute respiratory failure.^[^^13^^]^ In patients without cirrhosis, ARDS incidence has shown a gradual upward trend.^[^^14^^,^^15^^]^ Mortality ranges from 35% to 45%,^[^[14], [15], [16], [17]^]^ and has improved over recent decades.^[^^18^^]^ In contrast, early studies on ARDS reported ICU mortality rates up to 90% for patients with cirrhosis,^[^^19^^,^^20^^]^ contributing to the systematic exclusion of these patients from ARDS randomized controlled trials.^[^^21^^]^ As a result, data remain scarce and mostly retrospective: two small retrospective cohorts found a high ICU mortality rate in this population, up to 82%,^[^^22^^,^^23^^]^ while a more recent larger cohort reported a 57% 90-day mortality rate.^[^^24^^]^ Importantly, ARDS is frequently under-recognized in clinical practice.^[^^16^^]^ Whether similar under-recognition occurs in cirrhosis – a population with overlapping radiographic and hemodynamic abnormalities – remains largely unexplored. Likewise, the true prevalence of ARDS in patients with cirrhosis, the evolution of its outcomes over time, and the prognostic factors specific to this population remain insufficiently characterized, with no prospective data available.

Our primary objective was to assess 28-day mortality among ventilated patients with cirrhosis presenting with ARDS and to identify associated factors. Secondary objectives were to assess ARDS prevalence in this population, analyze changes in patients’ characteristics and mortality over the 2007–2021 period, and determine whether outcomes differed for patients with cirrhosis whose primary cause of ICU admission was acute respiratory failure.

## Methods

### Study design and patient selection

This retrospective observational study was performed in two ICUs in two tertiary centers of hepatology of the Greater Paris area in France, namely La Pitié-Salpêtrière (Paris) and Paul Brousse (Villejuif). All consecutive adult patients admitted to the two ICUs between 1 February 2007 and 31 December 2021, with a diagnosis code of cirrhosis (ICD-10: K703, K746) were retrospectively screened from electronic medical records. For each of these patients, full clinical and biological data were reviewed to confirm the diagnosis of cirrhosis and identify those who met the inclusion criteria. Because ARDS is frequently under-recognized in clinical practice,^[^^16^^]^ we did not rely on documented diagnoses in the medical record. Instead, full electronic and paper charts were manually reviewed to assess whether ARDS criteria were met, and two senior intensivists independently validated each case.

Patients were eligible if they (1) had confirmed cirrhosis based on histology when available, or on clinical (portal hypertension with ascites and/or esophageal varices, hepatic encephalopathy) or radiological criteria (liver dysmorphia with or without signs of portal hypertension^[^^10^^,^^22^^,^^25^^]^; (2) required invasive mechanical ventilation during ICU stay; and (3) fulfilled ARDS criteria^[^^13^^]^ at any time during their ICU stay. We applied the 2023 global ARDS definition retrospectively, but restricted inclusion to invasively ventilated patients to ensure homogeneous severity.^[^^13^^]^ Regarding ARDS diagnosis, a cardiogenic pulmonary edema was excluded based on clinical evaluation, echocardiography, and the absence of significant left ventricular dysfunction. Patients with a documented decision to limit or withdraw life-sustaining treatments within 24 h of ICU admission were excluded.

The study was approved by the ethics committee of the French Intensive Care Society (CE SRLF 22-075), which waived the need for informed consent from individual patients due to the retrospective nature of the study.

The study was reported in accordance with the Strengthening the Reporting of Observational Studies in Epidemiology (STROBE) guidelines, and the corresponding checklist is provided in Supplementary Table S1.

### Data collection

At each hospital, data were recorded from medical records. At the time of ICU admission, the following variables were recorded: date of hospital and ICU admission, age, sex, cause of cirrhosis and Child-Pugh score,^[^^26^^]^ Simplified Acute Physiologic Score II (SAPS II),^[^^27^^]^ Model for End-Stage Liver Disease (MELD),^[^^28^^]^ Acute on Chronic Liver Failure (ACLF),^[^^7^^]^ international normalized ratio (INR), platelet count, blood albumin, bilirubin, and creatinine. Primary reason for ICU admission was determined by two experienced physicians (Decavele and Demoule) and categorized as follows: acute respiratory failure, coma, gastrointestinal bleeding with shock, septic shock, acute renal failure, and other. ARDS onset was defined as the time point at which the patient first met ARDS criteria, based on clinical and radiological findings during the ICU stay. The risk factor for ARDS and ARDS severity (mild, moderate, severe) were also collected,^[^^13^^]^ with recording of the ratio of partial pressure of oxygen to fraction of inspired oxygen (PaO_2_/FiO_2_ ratio). During ICU stay, need for renal replacement therapy and vasopressors, prone positioning, use of neuromuscular blockers and nitric oxide were recorded. In case of documented infection, the identified pathogen was recorded. Finally, the following outcomes were assessed: 28-day and 90-day mortality after ARDS onset, duration of mechanical ventilation, ICU and hospital length of stay, and liver transplantation during the ICU stay or up to 90 days of follow-up.

### Statistical analysis

Quantitative variables are reported as median (interquartile range) and qualitative variables as number (percentage), as continuous variables were not assumed to be normally distributed. Continuous variables were compared using the Mann–Whitney *U* test, and categorical variables using the Chi-square test. Patients were followed until death or 90 days after diagnosis of ARDS. The primary outcome was 28-day mortality after the onset of ARDS to provide a consistent timeframe for outcome assessment and avoid immortal time bias. We used logistic regression to identify factors independently associated with 28-day mortality. Fixed-effect variables included in the model were age, sex, MELD score at admission (treated as a continuous variable), acute respiratory failure as the primary reason for ICU admission, and PaO_2_/FiO_2_ ratio at ARDS onset. Continuous predictors (age, MELD, PaO₂/FiO₂) were modeled on their continuous scales. Linearity on the logit scale was assessed using restricted cubic splines (df = 2) and compared with the linear specification via likelihood-ratio tests and Akaike information criterion (AIC)/ Bayesian information criterion (BIC); as splines did not materially improve fit (Supplementary Table S2), linear terms were retained for parsimony and interpretability. Collinearity was assessed using variance inflation factors. To assess robustness to unmeasured confounding, we reported E-values for each significant adjusted association, for protective effects (odds ratio [OR] < 1), E-values were computed on the reciprocal OR. Model performance and calibration were then summarized by the c-statistic (area under the curve (AUC) with bootstrap 95% confidence interval [CI]), the Brier score, and a calibration plot.

Sensitivity analysis were performed: (1) using a Firth penalized logistic regression, (2) re-defining the presentation variable by timing of ARDS (early ≤48 h and ≤72 h from ICU admission *vs.* later), replacing primary cause for ICU admission in the model, and (3) by adding to the model the period during which the patient was admitted to the ICU (before *vs.* after January 1, 2015).

To visually explore the effects of significant factors, Kaplan–Meier survival curves for mortality from ARDS diagnosis were generated and compared using absolute restricted mean survival time difference. These curves are descriptive and independent of the logistic regression analysis on 28-day mortality. Analyses were performed with R software version 4.0.3 (version 4.0.3, R Core Team 2020; https://www.R-project.org/,Vienna, Austria). No missing-data imputation was performed**.** The significance level for statistical tests was set at 0.05.

## Results

**Figure 1:**
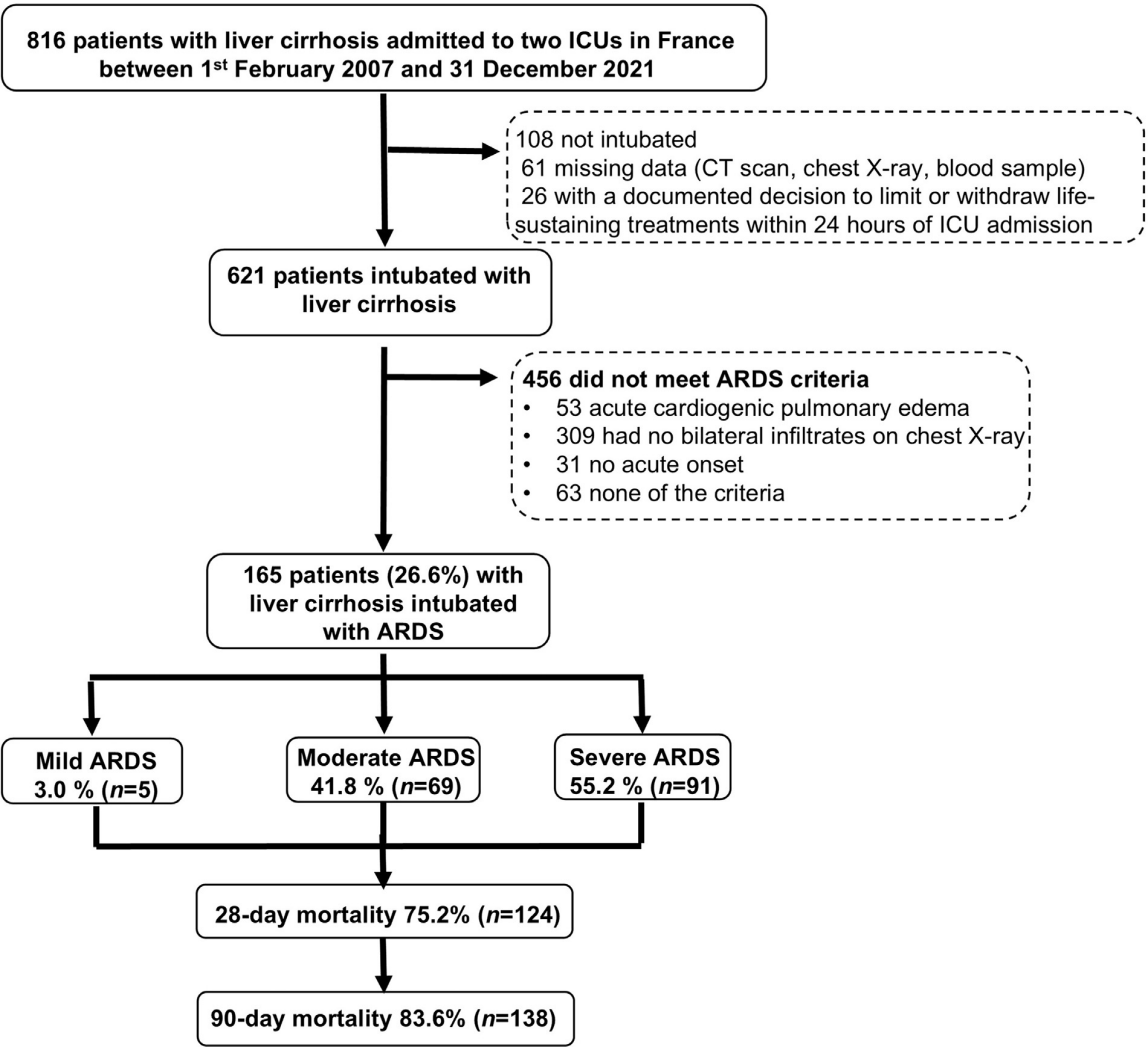
Flow chart of the study.

ARDS: Acute respiratory distress syndrome; CT: Computed tomography; ICU: Intensive care unit.

Figure 1 shows the study flow chart. Over the 15-year study period, 816 patients with cirrhosis were admitted to the two participating ICUs. One hundred and ninety-five were excluded because they were not intubated, had missing data or had a documented decision to limit or withdraw life-sustaining treatments within the first 24 h of ICU admission. Among the 621 intubated patients, 456 did not meet ARDS criteria. Eventually, 165 patients were included and analyzed. The prevalence of ARDS among intubated patients with cirrhosis was 26.6%. Eighty-nine (53.9%) patients were admitted during the 2007–2014 period, and 76 (46.1%) during the 2015–2021 period.

### Patient’s characteristics

Patients’ characteristics are depicted in Table 1. Patients were mostly men (73.9%), median aged 54 (IQR: 46–60) years. Cirrhosis was mainly due to alcohol-related liver disease (84.8%). The Child-Pugh class was C in 86.1% of patients. The primary cause of ICU admission was acute respiratory failure (52.1%), gastrointestinal bleeding with shock (18.2%), septic shock (15.8%), coma (6.1%), and acute renal failure (2.4%). Patients’ characteristics did not differ between the two study periods (Supplementary Table S3), nor did they differ between patients admitted for acute respiratory failure and other patients (Supplementary Table S4).

**Table 1:** Patients characteristics at admission to the ICU.

Characteristics	All patients (***n*** = 165)	28-day survivors (*n* = 41)	28-day non-survivors (*n* = 124)	*P* value
Age (years)	54 (46–60)	55 (45–59)	53 (46–60)	0.796
Sex (Female)	43 (26.1)	8 (19.5)	35 (28.2)	0.370
ICU length of stay (days)	12 (5–20)	21 (12–37)	10 (5–16)	<0.001
Period of admission				0.095
2007–2014	89 (53.9)	17 (41.5)	72 (58.1)	
2015–2021	76 (46.1)	24 (58.5)	52 (41.9)	
Cause of cirrhosis*				
Alcohol-related	140 (84.8)	38 (92.7)	102 (82.3)	0.173
Viral	18 (10.9)	6 (14.6)	12 (9.7)	0.553
Non-alcoholic fatty liver disease	19 (11.5)	5 (12.2)	14 (11.3)	>0.999
Other	9 (5.5)	0	9 (7.3)	0.168
Child Pugh score at ICU admission				0.225
A	3 (1.8)	1 (2.4)	2 (1.6)	
B	20 (12.1)	8 (19.5)	12 (9.7)	
C	142 (86.1)	32 (78.0)	110 (88.7)	
Score	12 (11–13)	12 (10–13)	12 (11–13)	0.066
Portal vein thrombosis	14 (8.5)	4 (9.8)	10 (8.1)	0.989
Hepatocellular carcinoma	9 (5.5)	1 (2.4)	8 (6.5)	0.559
Primary cause for ICU admission				0.297
Acute respiratory failure	86 (52.1)	28 (68.3)	58 (46.8)	
Coma	10 (6.1)	2 (4.9)	8 (6.5)	
Gastrointestinal bleeding with shock	30 (18.2)	5 (12.2)	25 (20.2)	
Septic shock	26 (15.8)	4 (9.8)	22 (17.7)	
Acute renal failure	4 (2.4)	1 (2.4)	3 (2.4)	
Other	9 (5.5)	1 (2.4)	8 (6.5)	

Data are expressed as median (interquartile range) for quantitative variables and as *n* (%) for qualitative variables.

ICU: Intensive care unit.

⁎ In a given patient, cirrhosis could have more than one cause.

### Severity and cirrhosis complications on ICU admission

On ICU admission, the median SAPS II, SOFA and MELD scores were 60 (IQR: 44–72), 13 (IQR: 11–14), and 29 (IQR: 23–36), respectively (Table 2). Most patients (68.5%) had grade 3 ACLF. The SAPS II score was lower during the 2015–2021 period compared to 2007–2014, while the MELD and SOFA scores remained unchanged (Supplementary Table S5). Regarding cirrhosis complications at ICU admission, 30.3% of patients presented with alcohol-related hepatitis, 81.2% with ascites, 27.9% with gastrointestinal bleeding, and 71.5% with hepatic encephalopathy (Table 2). Seventy-eight percent of patients presented more than one complication. INR was 2.8 (2.0–3.7), platelet count was 82 (54–127) × 10^9^/L, blood creatinine was 111 (65–205) µmol/L, and total serum bilirubin was 192 (92–352) µmol/L (Table 2). These variables did not differ between the two study periods (Supplementary Table S5). Patients admitted to the ICU for acute respiratory failure had lower SOFA and MELD scores at admission and were less likely to present with gastrointestinal bleeding as a complication of cirrhosis (Supplementary Table S6).

**Table 2:** Severity and cirrhosis complications at intensive care unit admission.

Characteristics	All patients (*n* = 165)	28-day survivors (*n* = 41)	28-day non-survivors (*n* = 124)	*P* value
Biology at ICU admission				
International normalized ratio	2.8 (2.0–3.7)	2.1 (1.8–2.8)	2.9 (2.2–3.9)	<0.001
Platelets(× 10^9^/L)	82 (54–127)	110 (61–200)	79 (51–111)	0.005
Serum creatinine(µmol/L)	111 (65–205)	75 (59–163)	117 (75–215)	0.058
Total serum bilirubin(µmol/L)	192 (92–352)	219 (46–474)	192 (98–335)	0.896
Albumin(g/L)	27 (22–31)	28 (25–31)	27 (22–31)	0.301
Severity at ICU admission				
SAPS II	60 (44–72)	54 (39–67)	61 (44–73)	0.146
SOFA	13 (11–14)	11 (10–13)	13 (11–14)	<0.001
MELD	29 (23–36)	24 (22–28)	32 (26–39)	<0.001
ACLF grade at ICU admission				0.193
No ACLF	4 (2.4)	2 (4.9)	2 (1.6)	
1	10 (6.1)	4 (9.8)	6 (4.8)	
2	38 (23.0)	12 (29.3)	26 (21.0)	
3	113 (68.5)	23 (56.1)	90 (72.6)	
Cirrhosis complication at ICU admission				
Acute alcoholic hepatitis	50 (30.3)	16 (39.0)	34 (27.4)	0.228
Ascites	134 (81.2)	31 (75.6)	103 (83.1)	0.407
Gastrointestinal bleeding	46 (27.9)	10 (24.4)	36 (29.0)	0.709
Hepatic encephalopathy	118 (71.5)	27 (65.9)	91 (73.4)	0.467
Liver transplantation				
On list before ICU admission	18 (10.9)	3 (7.3)	15 (12.1)	0.574
Joined the list during ICU stay	14 (8.5)	6 (14.6)	8 (6.5)	0.191
Transplantation during ICU stay	6 (3.6)	5 (12.2)	1 (0.8)	0.004
Transplantation within 90 days after ICU discharge	6 (3.6)	6 (14.6)	0 (0.0)	<0.001
ICU length of stay (days)	12 (5–20)	21 (12–37)	10 (5–16)	<0.001

Data are expressed as median (interquartile range) for quantitative variables and as *n*(%) for qualitative variables.

ACLF: Acute on chronic liver failure; ICU: Intensive care unit; MELD: Model for end-stage liver disease; SAPS II: Simplified acute physiology score; SOFA: Sepsis-related organ failure assessment.

Regarding liver transplantation, 10.9% of patients were already on the transplant list on ICU admission, 8.5% joined the list during the ICU stay (Table 2). There was an increase between the two study periods (1.1% in 2007–2014 *vs*. 17.1% in 2015–2021, *P* < 0.001) (Supplementary Table S5). Eventually, 12 patients received liver transplant, 6 during ICU stay and 6 after ICU discharge (Table 2).

### ARDS characteristics and management

Time from ICU admission to intubation was 0 (IQR: 0–1) days (Table 3). The median time from ICU admission to ARDS onset was 2 (IQR: 1–5) days (Table 3). It did not differ between the two study periods (Supplementary Table S7). It was shorter in patients whose primary cause of admission was acute respiratory failure (1 [0–3] day *vs*. 3 [1–6] days, *P* < 0.001) (Supplementary Table S8). Pneumonia was a risk factor for ARDS for 113 (68.5%) patients, of whom 65 (57.5%) had a microbiologically documented bacterial, viral, or fungal infection. Extrapulmonary sepsis was a risk factor for ARDS in 23.0% of patients. The median PaO_2_/FiO_2_ ratio at ARDS onset was 118 (IQR: 84–160) mmHg. ARDS severity was mild in 3.0% of patients, moderate in 41.8%, and severe in 55.2% (Table 3). Ninety-seven (63.8%) patients received neuromuscular blockers, 21.8% had at least one prone position session, and 25.8% received nitric oxide. Vasopressors were given to 97.6% and renal replacement therapy to 57.0% of patients (Table 3). ARDS characteristics and management did not differ between the two study periods, except for the use of prone positioning, which increased in 2015–2021 as compared to 2007–2014 (Supplementary Table S7). In patients admitted for acute respiratory failure, pneumonia was more likely to be the risk factor for ARDS than in patients admitted for another reason (Supplementary Table S8).

**Table 3:** Acute respiratory distress syndrome characteristics and management.

Characteristics	All patients (*n* = 165)	28-day survivors (*n* = 41)	28-day non-survivors (*n* = 124)	*P* value
Initial respiratory management and intubation				
Noninvasive ventilation	30 (18.2)	3 (7.3)	27 (21.8)	0.065
High-flow oxygen	10 (6.1)	1 (2.4)	9 (7.3)	0.457
Time from ICU admission to intubation (days)	0 (0–1)	0 (0–1)	0 (0–2)	0.018
Intubation criteria				0.942
Respiratory	126 (76.4)	32 (78.0)	94 (75.8)	
Endoscopy	27 (16.4)	6 (14.6)	21 (16.9)	
Neurology	12 (7.3)	3 (7.3)	9 (7.3)	
Time from ICU admission to ARDS onset (days)	2 (1–5)	1 (0–3)	2 (1–5)	0.026
Risk factors for ARDS				0.364
Pneumonia	113 (68.5)	31 (75.6)	82 (66.1)	
Extrapulmonary sepsis	38 (23.0)	5 (12.2)	33 (26.6)	
Blood transfusion	9 (5.5)	3 (7.3)	6 (4.8)	
Other risk factor	2 (1.2)	1 (2.4)	1 (0.8)	
No risk factor	3 (1.8)	1 (2.4)	2 (1.6)	
PaO_2_/FiO_2_ at ARDS onset	118 (84–160)	127 (100–180)	116 (81–155)	0.182
Worst PaO_2_/FiO_2_ during ICU stay	96 (69–122)	106 (84–140)	84 (67–117)	0.011
ARDS severity*				0.043
Mild	5 (3.0)	1 (2.4)	4 (3.2)	
Moderate	69 (41.8)	24 (58.5)	45 (36.3)	
Severe	91 (55.2)	16 (39.0)	75 (60.5)	
Organ support and ARDS management during ICU stay				
Renal replacement therapy	94 (57.0)	20 (48.8)	74 (59.7)	0.298
Vasopressor	161 (97.6)	39 (95.1)	122 (98.4)	0.553
Neuromuscular blockers†	97 (63.8)	23 (61.1)	74 (64.9)	0.722
Prone position‡	33 (21.8)	10 (25.7)	23 (20.2)	0.570
Nitric oxide‡	39 (25.8)	11 (28.3)	28 (25.1)	0.748

Data are expressed as median (interquartile range) for quantitative variables and as *n*(%) for qualitative variables.

ARDS: Acute respiratory distress syndrome; ICU: Intensive care unit; PaO_2_/FiO_2_: Partial pressure of arterial oxygen/fraction of inspired oxygen ratio.

⁎ Based on the worst PaO_2_/FiO_2_ ratio during ICU stay.

† The number of patients in this item was 152.

‡ The number of patients in this item was 151.

### 28-day all-cause mortality

The 28-day mortality was 75.2% (*n* = 124) and did not significantly change over time (80.9% in 2007–2014 *vs.* 68.4% in 2015–2021, *P* = 0.064) in univariate analysis (Supplementary Table S5). Ninety-day mortality was 83.6% (*n* = 138) and did not change over time (87.6% in 2007–2014 *vs.* 78.9% in 2015–2021, *P* = 0.132). Patients admitted for acute respiratory failure had a lower 28-day mortality than patients admitted for other causes (67.4% *vs.* 83.5%, *P* = 0.027) (Supplementary Table S6).

Tables 1, 2, and 3 outline identified factors associated with 28-day mortality. In the multivariate analysis, three factors were independently associated with 28-day mortality. A higher MELD score at ICU admission was significantly associated with increased mortality (odds ratio [OR]= 2.31, 95% confidence interval [CI]: 1.48 to 3.80, *P* < 0.001). Conversely, a higher PaO_2_/FiO_2_ ratio at ARDS diagnosis and ICU admission due to acute respiratory failure were independently associated with lower 28-day mortality (OR = 0.49, 95% CI: 0.24 to 0.97, *P* = 0.039; and OR = 0.44, 95% CI: 0.19 to 0.97, *P* = 0.047, respectively) (Table 4). For MELD, PaO₂/FiO₂, and primary cause for ICU admission, point-estimate E-values ranged from 2.2 to 2.4, indicating that an unmeasured confounder would need to be associated with both the exposure and the outcome by a risk ratio ≥2 to explain away the observed associations (Supplementary Table S9). The model achieved an AUC of 0.75 (95% CI: 0.68 to 0.84, bootstrap) with a Brier score of 0.157, and calibration was adequate with only minor deviations on the plot (Supplementary Figure S1).

**Table 4:** Factors associated with 28-day mortality in multivariable analysis.

Variables	Univariate OR (95% CI)	*P* value	Adjusted OR (95% CI)	*P* value
Age, per year increase	1.01 (0.98–1.04)	0.572	1.02 (0.98–1.05)	0.336
Sex (Male)	1.62 (0.71–4.08)	0.273	1.97 (0.79–5.37)	0.162
MELD score at ICU admission, per 10-point increase	2.32 (1.51–3.72)	< 0.001	2.31 (1.48–3.80)	< 0.001
PaO_2_/FiO_2_ ratio at ARDS diagnosis, per 100-point increase	0.63 (0.33–1.18)	0.146	0.49 (0.24–0.97)	0.039
ICU admission for acute respiratory failure	0.41 (0.19–0.85)	0.019	0.44 (0.19–0.97)	0.047

OR and 95% CI were computed using univariate and multivariate logistic regression.

ARDS: Acute respiratory distress syndrome; CI: Confidence intervals; ICU: Intensive care unit; MELD: Model for end-stage liver disease; OR: Odds ratios; PaO_2_/FiO_2__:_ Partial pressure of arterial oxygen/fraction of inspired oxygen rate.

Sensitivity analyses using Firth penalized logistic regression and redefining the presentation variable by ARDS timing (early ≤48 h or ≤72 h from ICU admission *vs.* later) produced results consistent with the primary model (Supplementary Tables S10, S11, and S12). Sensitivity analysis, which included the admission period (2007–2014 *vs.* 2015–2021) as a covariate, yielded similar results, the admission period being not significantly associated with 28-day mortality (OR=1.69, 95% CI: 0.77 to 3.78, *P* = 0.192) (Supplementary Table S13).

**Figure 2:**
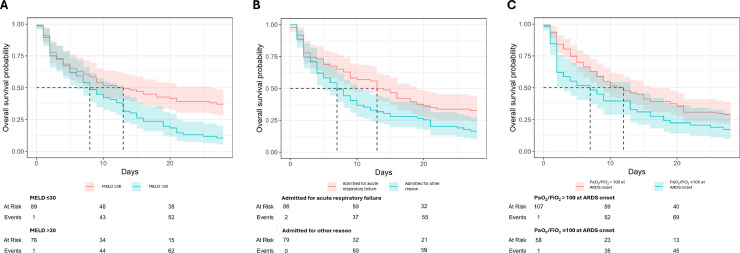
Cumulative survival up to 28 days from ARDS onset according to MELD score (A) on admission (<30 *vs*. ≥30), ICU admission reason (B, admitted for acute respiratory failure *vs.* admitted for other reasons), and PaO_2_/FiO_2_ ratio (C, ≤100 mmHg *vs*. >100 mmHg) at ARDS onset.

ARDS: Acute respiratory distress syndrome; ICU: Intensive care unit; MELD: Model for end-stage liver disease; PaO_2_/FiO_2_: Partial pressure of arterial oxygen/fraction of inspired oxygen ratio.

Figure 2 illustrates cumulative survival probability up to 28 days, stratified by MELD score at ICU admission, reason for ICU admission, and PaO_2_/FiO_2_ ratio at ARDS onset.

### Documented infection

An infection was documented in 87 patients (52.3%) (Supplementary Table S14). Gram-negative bacilli accounted for 49.4% of all documented infections, whereas Gram-positive cocci, fungal, and viral were identified in 21.8%, 18.4%, and 9.2% of all documented infections, respectively. Viral infection, such as SARS-CoV-2 infection, was only responsible for ARDS with pneumonia as a risk factor. *Enterococcus* was documented in 7 (31.8%) ARDS with extra-pulmonary sepsis as a risk factor.

## Discussion

The main findings of our study are as follows. First, mortality in patients with cirrhosis requiring mechanical ventilation and presenting with ARDS is very high, consistent with prior studies.^[^[22], [23], [24]^]^ Second, ARDS is frequent in this population, affecting 26.6% of patients. Third, while some markers of severity at ICU admission (SAPS II) improved over the 15-year study period, overall mortality did not significantly decrease. Fourth, patients with higher MELD scores, lower PaO₂/FiO₂ ratios at ARDS onset, and those admitted for causes other than acute respiratory failure are at significantly higher risk of death, highlighting distinct patient profiles. These findings address our primary objective of identifying prognostic factors for short-term mortality and also our secondary objectives, including ARDS prevalence, temporal changes in severity and outcomes, and the impact of admission diagnosis on prognosis.

The characteristics of our population are in line with those of previous cohorts of patients with cirrhosis managed in the ICU, *i.e.*, 50- to 60-year-old men, whose main cause of cirrhosis is excessive alcohol consumption.^[^^9^^,^^10^^,^[22], [23], [24]^]^ In these studies, ICU mortality observed in patients with cirrhosis requiring mechanical ventilation is high.^[^^9^^,^^10^^]^ We observed a 28-day mortality of 75.2%, and a 90-day mortality of 83.6%. In general ARDS cohorts, mortality is lower, as it ranges from 35% to 45%.^[^^16^^,^^29^^,^^30^^]^ To date, only three studies have specifically investigated ARDS in patients with cirrhosis.^[^[22], [23], [24]^]^ Two reported a 90-day mortality of around 60%,^[^^22^^,^^24^^]^ while another, based on similar selection criteria to ours, found a mortality close to 80%.^[^^23^^]^ Differences in reported outcomes across these studies likely reflect variations in study design and patients selection. Two studies identified patients with cirrhosis within broader ARDS cohorts,^[^^22^^,^^24^^]^ thereby mostly including patient admitted for acute respiratory failure, in whom we observed a 67.4% 28-day mortality in the present study. This supports that cirrhosis itself likely contributes to worse outcomes beyond acute illness severity. In contrast, a study that identified ARDS within broader cohorts of patients with cirrhosis,^[^^19^^]^ such as ours, also captured patients initially admitted for another reason than acute respiratory failure, who developed ARDS secondarily during their ICU stay – a subgroup known with higher mortality in our study. This likely explains the higher overall mortality observed in our cohort. Another possible explanation for these differences is the higher severity of patients in our study, as indicated by the elevated SAPS II scores and distinct ARDS risk factors, along with a higher pneumonia rate compared to other studies.^[^^22^^,^^24^^]^

We found that the prevalence of ARDS among ventilated patients with cirrhosis was 26.6%, three times more than what was observed in a smaller cohort study conducted in a liver transplant center in the US.^[^^23^^]^ Although this may reflect different profiles of patients with cirrhosis admitted to the ICU, it may also suggest under-recognition of ARDS in this population. ARDS under-recognition has been very well evidenced in the LUNG SAFE cohort, where clinicians failed to recognize ARDS in approximately 40% of patients.^[^^16^^]^ This has important implications in terms of patient management. Indeed, ARDS patients require lung protective ventilation, including low tidal volume and, depending on the severity of hypoxemia and respiratory system mechanics and morphology, high positive end-expiratory pressure and prone positioning.^[^^31^^]^ These treatments are associated with better outcomes and failing to apply them may alter patients prognosis.^[^^31^^]^

Our study found a significantly lower SAPS II during the 2015–2021 period. Although mortality did not significantly differ between the two study periods, a trend toward improved outcome was observed, with mortality decreasing from 80.9% in 2007–2014 to 68.4% in 2015–2021 (*P* = 0.064). Additionally, there was a trend toward a higher PaO_2_/FiO_2_ ratio at ARDS onset in the latter period. This may suggest that, over the 15-year study period, patients with cirrhosis got an easier and earlier access to the ICU. These data are consistent with those showing an increase in the rate of admission of patients with cirrhosis in ICUs in the United Kingdom.^[^^3^^]^ Over the last few years, publications have reported an overall improvement in the prognosis of patients with cirrhosis in the ICU.^[^^3^^,^^32^^,^^33^^]^ The lack of improvement in mortality that we observed suggests that this is not the case for patients with cirrhosis presenting with ARDS. This is consistent with a recent report, which found no improvement in the prognosis of patients with cirrhosis presenting with ARDS over an 18-year period.^[^^24^^]^

Three factors were independently associated with 28-day mortality. As this was a retrospective observational study, these associations should not be interpreted as causal. The MELD score being validated as a predictor of survival in patients with cirrhosis, it is not surprising that we found an independent relationship between this score and 28-day mortality in our cohort.^[^^28^^]^ This is also the case of PaO_2_/FiO_2_, which is associated with mortality in ARDS patients.^[^^16^^]^ An interesting and novel finding was that patients admitted for acute respiratory failure had lower mortality. It may suggest two different patient profiles. First, patients admitted for acute respiratory failure who rapidly develop an ARDS. Second, patients admitted for a complication of cirrhosis – such as gastrointestinal bleeding with shock, septic shock, or coma with aspiration – who subsequently develop ARDS during their ICU stay. The mortality rate for patients in the first profile is 67.4% and 83.5% in the second. While causal inference cannot be made in this retrospective study, recognizing these patterns may help tailor treatment intensity and avoid disproportionate interventions in patients with extremely poor expected outcomes.

Our analysis revealed that, during the second study period (2015–2021), a greater number of patients were registered on the transplant list within their ICU stay, with a trend towards an increased number of transplants being performed during ICU stay during the second study period, indicating a shift in clinical management for these critically ill patients. However, numbers remained low due to frequent contraindications in ICU patients. As transplantation is the only curative treatment for cirrhosis, this underscores the poor prognosis once ARDS develops and highlights the potential benefit of earlier evaluation and listing before critical illness.

Beyond their prognostic implications, these results raise important ethical considerations regarding ICU admission and treatment intensity in end-stage cirrhosis. The very high mortality observed suggests that, for some patients, escalation to invasive mechanical ventilation may offer limited benefit and should prompt early multidisciplinary discussions about goals of care and potential futility. Conversely, identifying patients with a realistic chance of recovery – for instance, those eligible for liver transplantation – remains crucial to avoid undue therapeutic limitation.

Strengths of this study include its bicentric design and large sample size. To the best of our knowledge, this is the largest cohort of patients with cirrhosis and ARDS reported to date, and the first to identify independent prognostic factors for short-term mortality in this specific population. By highlighting the respective contributions of hepatic dysfunction (MELD), respiratory severity (PaO₂/FiO₂), and ICU admission cause, our results help reconcile discrepancies among prior, smaller cohorts.^[^[22], [23], [24]^]^ Our study has several limitations. First, it was a retrospective study, with inherent risks of missing data and information bias. Because ARDS is frequently under-recognized in ICU clinical practice, as shown in the LUNG SAFE study,^[^^16^^]^ inclusion required full manual review of each chart rather than reliance on reported diagnoses. Although this approach reduces misclassification, it also illustrates the labor-intensive nature of retrospective ARDS identification. To minimize this, patient selection was performed systematically using CIM-10 coding followed by independent validation of ARDS and cirrhosis diagnosis by two senior intensivists. Second, we included all patients with cirrhosis presenting with ARDS, regardless of the initial reason for ICU admission. To mitigate the risk of immortal time bias, we analyzed 28-day mortality from ARDS onset rather than from ICU admission, but it does not eliminate survivor selection bias. To our knowledge, this is the first study to use this endpoint in this population, enabling a more precise assessment of patients who developed ARDS during their ICU stay. Third, we did not collect ventilatory data, such as positive end-expiratory pressure level, tidal volume, and respiratory system plateau pressure, as these were not consistently available in the medical records over the 15-year retrospective study period. We acknowledge that this prevents a direct assessment of adherence to lung-protective ventilation, which should be considered when interpreting our findings. Fourth, although ARDS was retrospectively defined using the 2023 global criteria,^[^^13^^]^ we restricted inclusion to intubated patients to ensure a homogeneous severity profile. As a result, our findings apply specifically to invasively ventilated ARDS in cirrhosis and may not extend to the broader spectrum of non-intubated ARDS now encompassed in the updated definition. Fifth, despite adjustment, residual confounding remains possible. However, our E-values suggest that an unmeasured confounder would need a risk-ratio ≥2 with both exposure and outcome to fully explain away the observed effects. Liver transplantation before death is a potential competing event, but it was rare during ICU in our cohort, making any substantial impact on population-level mortality unlikely. Sixth, detailed data on advanced ARDS therapies were limited. Data on prone positioning were available for 152 patients, of whom only 33 (21.6%) received at least one session – 6.7% during 2007–2014 and 35.5% during 2015–2021 – reflecting an increase over time, following the publication of the results of the PROSEVA trial.^[^^34^^]^ Importantly, during the second period, the median PaO₂/FiO₂ ratio was 127 mmHg (IQR 98–172), indicating that roughly two-thirds of patients met the severity threshold for prone positioning (PaO₂/FiO₂ <150). Among these patients, more than half received prone positioning, which is higher than the approximately 32% reported in the LUNG SAFE cohort among patients with comparable PaO₂/FiO₂ severity[ 16]. The remaining gap during the most recent period may be explained by evolving local practices, patient-specific contraindications, or clinical factors related to cirrhosis, such as hemodynamic instability, coagulopathy, or severe ascites, which may limit the feasibility of prone positioning. Under-recognition of ARDS may also have contributed to the limited use of prone positioning. ECMO was rarely used, and detailed data were not systematically collected. Finally, due to the high mortality rate, the results of the multivariate analysis should be interpreted with caution; nonetheless, the results were robust across sensitivity analyses.

## Conclusions

ARDS affects more than one in four intubated patients with cirrhosis and remains associated with extremely high mortality. Over the past 15 years, outcomes have not improved despite changes in ICU practices. Three factors independently predicted 28-day mortality: hepatic dysfunction (MELD score), respiratory severity (PaO₂/FiO₂ ratio), and reason for ICU admission. Patients admitted for acute respiratory failure have a better survival than those developing ARDS secondary to other complications, highlighting distinct clinical profiles that may help guide triage and treatment intensity. Systematic screening for ARDS in patients with cirrhosis admitted in the ICU could help reduce under-recognition and promote the application of lung-protective ventilation strategies. Further research should focus on identifying phenotypes and management pathways associated with improved outcomes and on defining the role of early liver transplantation evaluation in this high-risk group.

## Acknowledgements

Naima Zemirli and Juliette Blondi, clinical research manager; and Laura Wakselman, study coordinator in the Clinical Research Unit of La Pitié-Salpêtriére in Paris

## Funding

This research did not receive any specific grant from funding agencies in the public, commercial, or not-for-profit sectors.

## Ethics Statement

The study was approved by the ethics committee of the French Intensive Care Society (CE SRLF 22-075), which waived the need for informed consent from individual patients due to the retrospective nature of the study.

## Conflict of Interest

Adam Celier, Marie-Amélie Ordan, Aymeric Lanore, Julien Mayaux, Philippe Ichaï: no conflict of interest to declare. Marika Rudler reports speaker fees from Gore, Abbvie, outside the submitted work. Maxens Decavèle reports congress registration fees from ISIS Medical and SOS Oxygène, outside the submitted work. Alexandre Demoule reports grants from the French Ministry of Health, Lungpacer, Respinor, Liberate Medical; consulting fees from Respinor, Liberate Medical, SAT Lutech; payment or honoraria for lectures/presentations from Fisher & Paykel, Astra; support for attending meetings and/or travel from Respinor, outside the submitted work.

## Data Availability

The datasets used and/or analyzed during the current study are available from the corresponding author on reasonable request.

## CRediT authorship contribution statement

**Adam Celier:** Writing – review & editing, Writing – original draft, Visualization, Methodology, Formal analysis, Data curation, Conceptualization. **Marie-Amélie Ordan:** Writing – review & editing, Data curation, Conceptualization. **Aymeric Lanore:** Writing – review & editing, Software, Formal analysis. **Julien Mayaux:** Writing – review & editing, Data curation. **Philippe Ichaï:** Writing – review & editing, Validation, Investigation. **Marika Rudler:** Writing – review & editing, Validation, Investigation. **Maxens Decavèle:** Writing – review & editing, Validation, Supervision, Project administration, Methodology, Conceptualization. **Alexandre Demoule:** Writing – review & editing, Validation, Supervision, Project administration, Methodology, Investigation.

## Appendix Supplementary materials

Image, application 1
