# Supplementary material for: Characteristics and prognosis of patients with cirrhosis presenting with acute respiratory distress syndrome: A bicentric retrospective study
Source: J Intensive Med. 2026 Feb 20;6(4):365–73. doi: 10.1016/j.jointm.2025.12.008 (PMC13323546; doi:10.1016/j.jointm.2025.12.008)
Supplement: Supplementary file 1 [file mmc1.docx]

**Characteristics and prognosis of patients with cirrhosis presenting with acute respiratory distress syndrome: a bicentric retrospective study**

Adam Celier^1^, Marie-Amélie Ordan^2^, Aymeric Lanore^3,4^, Julien Mayaux^1^, Philippe Ichaï^2^, Marika Rudler^5,6^, Maxens Decavèle^1,7^, Alexandre Demoule^1,7^

**Online supplement**

This file provides additional methodological details, including model diagnostics and sensitivity analyses. To limit overfitting, the multivariable logistic model was restricted to five prespecified predictors, yielding an events-per-variable ratio of approximately 8. Linearity of continuous variables was assessed using restricted cubic splines; no improvement in fit was observed (Table S2). Internal bootstrap validation (200 resamples) showed acceptable discrimination (AUC 0.75; 95% CI 0.68–0.84) and good calibration (Figure S1).

Robustness was confirmed across sensitivity analyses, including Firth penalized logistic regression, alternative definitions of early ARDS (≤48 h and ≤72 h), and inclusion of the study period as a covariate (Tables S10–S13). Effect sizes remained consistent. E-values (Table S9) indicate that substantial unmeasured confounding would be required to fully explain the observed associations.

**Table S1. STROBE Statement—Checklist of items included in our study**

**Table S2. Linearity Assessment of Continuous Predictors**

**Table S3. Patients characteristics at admission to the intensive care unit, comparison between the two study periods 2007-2014 and 2015-2021**

**Table S4. Patients characteristics at admission to the intensive care unit, comparison between patients admitted to the intensive care unit for acute respiratory failure and those admitted for other reasons**

**Table S5. Severity and cirrhosis complications on intensive care unit admission, comparison between the two study periods 2007-2014 and 2015-2021**

**Table S6. Severity and cirrhosis complications on intensive care unit admission, comparison between patients admitted to the intensive care unit for acute respiratory failure and those admitted for other reasons**

**Table S7. Acute respiratory distress syndrome characteristics and management, comparison between the two study periods 2007-2014 and 2015-2021**

**Table S8. Acute respiratory distress syndrome characteristics and management, comparison between patients admitted to the intensive care unit for acute respiratory failure and those admitted for other reasons**

**Table S9. E-values for significant factors**

**Table S10. Sensitivity analysis: Firth penalized logistic regression for 28-day mortality**

**Table S11. Sensitivity analysis: Logistic regression for 28-day mortality with ARDS onset within 48 hours of ICU admission**

**Table S12. Sensitivity analysis: Logistic regression for 28-day mortality with ARDS onset within 72 hours of ICU admission**

**Table S13. Sensitivity analysis: Logistic regression for 28-day mortality adding period of admission 2007–2014**

**Table S14. Documented infections**

**Figure S1. Calibration of the multivariable logistic model at day 28**

**Table S1. STROBE Statement—Checklist of items included in our study.**

|  | Item No | Recommendation | Reported on page/section |
| --- | --- | --- | --- |
| **Title and abstract** | 1 | (*a*) Indicate the study’s design with a commonly used term in the title or the abstract | **Title** and **abstract** |
|  |  | (*b*) Provide in the abstract an informative and balanced summary of what was done and what was found | **Abstract** |
| Introduction | | |  |
| Background/rationale | 2 | Explain the scientific background and rationale for the investigation being reported | **Introduction** |
| Objectives | 3 | State specific objectives, including any prespecified hypotheses | **Introduction** |
| Methods | | |  |
| Study design | 4 | Present key elements of study design early in the paper | **Patients and Methods**  Study design and patients selection |
| Setting | 5 | Describe the setting, locations, and relevant dates, including periods of recruitment, exposure, follow-up, and data collection | **Patients and Methods**  Study design and patients selection |
| Participants | 6 | (*a*) Give the eligibility criteria, and the sources and methods of selection of participants. Describe methods of follow-up | **Patients and Methods**  Study design and patients selection |
|  |  | (*b*) For matched studies, give matching criteria and number of exposed and unexposed |  |
| Variables | 7 | Clearly define all outcomes, exposures, predictors, potential confounders, and effect modifiers. Give diagnostic criteria, if applicable | **Patients and Methods**  Data collection |
| Data sources/ measurement | 8 | For each variable of interest, give sources of data and details of methods of assessment (measurement). Describe comparability of assessment methods if there is more than one group | **Patients and Methods**  Data collection |
| Bias | 9 | Describe any efforts to address potential sources of bias | **Patients and Methods**  Statistical analysis |
| Study size | 10 | Explain how the study size was arrived at | Non applicable |
| Quantitative variables | 11 | Explain how quantitative variables were handled in the analyses. If applicable, describe which groupings were chosen and why | **Patients and Methods**  Statistical analysis |
| Statistical methods | 12 | (*a*) Describe all statistical methods, including those used to control for confounding | **Patients and Methods**  Statistical analysis |
|  |  | (*b*) Describe any methods used to examine subgroups and interactions | **Patients and Methods**  Statistical analysis |
|  |  | (*c*) Explain how missing data were addressed | **Patients and Methods**  Statistical analysis |
|  |  | (*d*) If applicable, explain how loss to follow-up was addressed |  |
|  |  | (*e*) Describe any sensitivity analyses | **Patients and Methods**  Statistical analysis |
| Results | | |  |
| Participants | 13 | (a) Report numbers of individuals at each stage of study—eg numbers potentially eligible, examined for eligibility, confirmed eligible, included in the study, completing follow-up, and analysed | **Results**  **Fig 1** |
|  |  | (b) Give reasons for non-participation at each stage | **Results**  **Fig 1** |
|  |  | (c) Consider use of a flow diagram | **Fig 1** |
| Descriptive data | 14 | (a) Give characteristics of study participants (eg demographic, clinical, social) and information on exposures and potential confounders | **Results**  Patient’s characteristics |
|  |  | (b) Indicate number of participants with missing data for each variable of interest | **Tables** |
|  |  | (c) Summarise follow-up time (eg, average and total amount) | Non applicable |
| Outcome data | 15 | Report numbers of outcome events or summary measures over time | Non applicable |
| Main results | 16 | (*a*) Give unadjusted estimates and, if applicable, confounder-adjusted estimates and their precision (eg, 95% confidence interval). Make clear which confounders were adjusted for and why they were included | **Results**  28-day all-cause mortality |
|  |  | (*b*) Report category boundaries when continuous variables were categorized | Non applicable |
|  |  | (*c*) If relevant, consider translating estimates of relative risk into absolute risk for a meaningful time period | Non applicable |
| Other analyses | 17 | Report other analyses done—eg analyses of subgroups and interactions, and sensitivity analyses | **Results**  28-day all-cause mortality |
| Discussion | | |  |
| Key results | 18 | Summarise key results with reference to study objectives | **Discussion** |
| Limitations | 19 | Discuss limitations of the study, taking into account sources of potential bias or imprecision. Discuss both direction and magnitude of any potential bias | **Discussion** |
| Interpretation | 20 | Give a cautious overall interpretation of results considering objectives, limitations, multiplicity of analyses, results from similar studies, and other relevant evidence | **Discussion** |
| Generalisability | 21 | Discuss the generalisability (external validity) of the study results | **Discussion** |
| Other information | | |  |
| Funding | 22 | Give the source of funding and the role of the funders for the present study and, if applicable, for the original study on which the present article is based | **Funding** |

**Table S2. Linearity Assessment of Continuous Predictors**

| **Predictor tested (df=2)** | **ΔDeviance** | **Δdf** | **LRT p-value** |
| --- | --- | --- | --- |
| **Age** | 0.256 | 1 | 0.613 |
| **MELD** | 0.012 | 1 | 0.913 |
| **PaO₂/FiO₂** | 0.022 | 1 | 0.882 |

Linearity of continuous predictors was evaluated with restricted cubic splines (df=2) and compared with linear terms using likelihood-ratio tests (LRT) on the same dataset. ΔDeviance and Δdf denote the change in model deviance and degrees of freedom versus the linear specification. No spline model materially improved fit (all p>0.60); therefore, linear terms were retained for parsimony and interpretability.

**Table S3. Patients characteristics at admission to the intensive care unit, comparison between the two study periods 2007-2014 and 2015-2021**

| **Characteristics** | **Period**  **2007-2014**  **(n=89)** | **Period**  **2015-2021**  **(n=76)** | ***P*-value** |
| --- | --- | --- | --- |
| Age (years) | 53 (46–60) | 54 (46–60) | 0.731 |
| Woman gender | 18 (20.2) | 25 (32.9) | 0.065 |
| Cause of cirrhosis^*^ |  |  |  |
| Alcohol-related | 73 (82.0) | 67 (88.2) | 0.273 |
| Viral | 11 (12.4) | 7 (9.2) | 0.518 |
| Non-alcoholic fatty liver disease | 11 (12.4) | 8 (10.5) | 0.713 |
| Other | 5 (5.6) | 4 (5.3) | >0.999 |
| Child Pugh score at ICU admission |  |  | 0.122 |
| A | 1 (1.1) | 2 (2.6) |  |
| B | 7 (7.9) | 13 (17.1) |  |
| C | 81 (91.0) | 61 (80.3) |  |
| Score | 12 (11–13) | 12 (10–13) | 0.484 |
| Portal vein thrombosis | 8 (9.0) | 6 (7.9) | 0.802 |
| Hepatocellular carcinoma | 6 (6.7) | 3 (3.9) | 0.508 |
| Primary cause for ICU admission |  |  | 0.486 |
| Acute respiratory failure | 43 (48.3) | 43 (56.6) |  |
| Coma | 6 (6.7) | 4 (5.3) |  |
| Gastrointestinal bleeding with shock | 21 (23.6) | 9 (11.8) |  |
| Septic shock | 13 (14.6) | 13 (17.1) |  |
| Acute renal failure | 2 (2.2) | 2 (2.6) |  |
| Other | 4 (4.5) | 5 (6.6) |  |

Data are expressed as median (interquartile range) for quantitative variables and as *n*(%) for qualitative variables.

ICU, intensive care unit.

^*^In a given patient, cirrhosis could have more than one cause.

**Table S4. Patients characteristics at admission to the intensive care unit, comparison between patients admitted to the intensive care unit for acute respiratory failure and those admitted for other reasons**

| **Characteristics** | **Admitted for acute respiratory failure**  **(n=86)** | **Admitted for other cause**  **(n=79)** | ***P*-value** |
| --- | --- | --- | --- |
| Age (years) | 53 (45–59) | 54 (47–60) | 0.546 |
| Woman gender | 27 (31.4) | 19 (24.1) | 0.699 |
| Length of stay in ICU, *days* | 12 (5–22) | 12 (7–17) | 0.589 |
| Cause of cirrhosis^*^ |  |  |  |
| Alcohol-related | 71 (82.6) | 69 (87.3) | 0.523 |
| Viral | 13 (15.1) | 5 (6.3) | 0.119 |
| Non-alcoholic fatty liver disease | 8 (9.3) | 11 (13.9) | 0.493 |
| Other | 5 (5.8) | 4 (5.1) | >0.999 |
| Child Pugh score at ICU admission |  |  | 0.840 |
| A | 2 (2.3) | 1 (1.3) |  |
| B | 11 (12.8) | 9 (11.4) |  |
| C | 73 (84.9) | 69 (87.3) |  |
| Score | 12 (11–13) | 12 (10–13) | 0.262 |
| Portal vein thrombosis | 9 (10.5) | 5 (6.3) | 0.501 |
| Hepatocellular carcinoma | 4 (4.7) | 5 (6.3) | 0.896 |

Data are expressed as median (interquartile range) for quantitative variables and as *n*(%) for qualitative variables.

ICU, intensive care unit.

^*^In a given patient, cirrhosis could have more than one cause.

**Table S5. Severity and cirrhosis complications on intensive care unit admission, comparison between the two study periods 2007-2014 and 2015-2021**

| **Characteristics** | **Period**  **2007-2014**  **(n=89)** | **Period**  **2015-2021**  **(n=76)** | ***P*-value** |
| --- | --- | --- | --- |
| Biology at ICU admission |  |  |  |
| International normalized ratio | 2.8 (2.0–3.7) | 2.8 (2.0–3.7) | 0.728 |
| Platelets(x10^9^/L) | 81 (52–116) | 83 (61–133) | 0.193 |
| Serum creatinine(µmol/L) | 121 (67–243) | 102 (63–159) | 0.226 |
| Total serum bilirubin(µmol/L) | 183 (72–341) | 212 (125–371) | 0.081 |
| Albumin(g/L) | 25 (21–29) | 28 (24–34) | 0.013 |
| Severity at ICU admission |  |  |  |
| SAPS II | 63 (47–75) | 53 (40–66) | 0.017 |
| SOFA | 13 (11–14) | 12 (11–14) | 0.353 |
| MELD | 31 (24–40) | 29 (23–35) | 0.142 |
| ACLF grade at ICU admission |  |  | 0.670 |
| No ACLF | 2 (2.2) | 2 (2.6) |  |
| 1 | 4 (4.5) | 6 (7.9) |  |
| 2 | 23 (25.8) | 15 (19.7) |  |
| 3 | 60 (67.4) | 53 (69.7) |  |
| Cirrhosis complication at ICU admission |  |  |  |
| Acute alcoholic hepatitis | 24 (27.0) | 26 (34.2) | 0.313 |
| Ascites | 73 (82.0) | 61 (80.3) | 0.773 |
| Gastro intestinal bleeding | 27 (30.3) | 19 (25.0) | 0.446 |
| Hepatic encephalopathy | 67 (75.3) | 51 (67.1) | 0.246 |
| Liver transplantation |  |  |  |
| On list before ICU admission | 11 (12.4) | 7 (9.2) | 0.518 |
| Joined the list during ICU stay | 1 (1.1) | 13 (17.1) | <0.001 |
| Transplantation during ICU stay | 1 (1.1) | 5 (6.6) | 0.096 |
| Transplantation within 90 days after ICU discharge | 3 (3.4) | 3 (3.9) | 0.272 |
| Mortality |  |  |  |
| 28-day | 72 (80.9) | 52 (68.4) | 0.064 |
| 90-day | 78 (87.6) | 60 (78.9) | 0.132 |

Data are expressed as median (interquartile range) for quantitative variables and as *n*(%) for qualitative variables.

ICU, intensive care unit; SAPS II, simplified acute physiology score; SOFA, sepsis-related organ failure assessment; MELD, model for end-stage liver disease; ACLF, acute on chronic liver failure.

**Table S6. Severity and cirrhosis complications on intensive care unit admission, comparison between patients admitted to the intensive care unit for acute respiratory failure and those admitted for other reasons**

| **Characteristics** | **Admitted for acute respiratory failure**  **(n=86)** | **Admitted for other cause**  **(n=79)** | ***P*-value** |
| --- | --- | --- | --- |
| Biology at ICU admission |  |  |  |
| International normalized ratio | 2.3 (1.9–3.6) | 2.9 (2.4–4.0) | 0.011 |
| Platelets(x10^9^/L) | 89 (60–149) | 79 (53–110) | 0.131 |
| Serum creatinine(µmol/L) | 82 (59–154) | 145 (82–256) | <0.001 |
| Total serum bilirubin(µmol/L) | 220 (97–373) | 183 (88–345) | 0.737 |
| Albumin(g/L) | 27 (23–31) | 27 (21–31) | 0.947 |
| Severity at ICU admission |  |  |  |
| SAPS II | 60 (38–72) | 60 (48–73) | 0.154 |
| SOFA | 12 (10–13) | 14 (12–16) | <0.001 |
| MELD | 28 (23–33) | 32 (26–39) | 0.013 |
| ACLF grade at ICU admission |  |  | 0.709 |
| No ACLF | 2 (2.3) | 2 (2.5) |  |
| 1 | 7 (8.1) | 3 (3.8) |  |
| 2 | 19 (22.1) | 19 (24.1) |  |
| 3 | 58 (67.4) | 55 (69.6) |  |
| Cirrhosis complication at ICU admission |  |  |  |
| Acute alcoholic hepatitis | 30 (34.9) | 20 (25.3) | 0.243 |
| Ascites | 69 (80.2) | 65 (82.3) | 0.891 |
| Gastro intestinal bleeding | 10 (11.6) | 36 (45.6) | <0.001 |
| Hepatic encephalopathy | 58 (67.4) | 60 (75.9) | 0.300 |
| Liver transplantation |  |  |  |
| On list before ICU admission | 12 (14.0) | 6 (7.6) | 0.290 |
| Joined the list during ICU stay | 9 (10.5) | 5 (6.3) | 0.501 |
| Transplantation during ICU stay | 4 (4.7) | 2 (2.5) | 0.756 |
| Transplantation within 90 days after ICU discharge | 5 (5.8) | 1 (1.3) | 0.142 |
| Mortality |  |  |  |
| 28-day | 58 (67.4) | 66 (83.5) | 0.027 |
| 90-day | 69 (80.2) | 69 (87.3) | 0.307 |

Data are expressed as median (interquartile range) for quantitative variables and as *n*(%) for qualitative variables.

ICU, intensive care unit; SAPS II, simplified acute physiology score; SOFA, sepsis-related organ failure assessment; MELD, model for end-stage liver disease; ACLF, acute on chronic liver failure.

**Table S7. Acute respiratory distress syndrome characteristics and management, comparison between the two study periods 2007-2014 and 2015-2021**

| **Characteristics** | **Period**  **2007-2014**  **(n=89)** | **Period**  **2015-2021**  **(n=76)** | ***P*-value** |
| --- | --- | --- | --- |
| Initial respiratory management and intubation |  |  |  |
| Noninvasive ventilation | 17 (19.1) | 13 (17.1) | 0.740 |
| High-flow oxygen | 5 (5.6) | 5 (6.6) | >0.999 |
| Intubation criteria |  |  | 0.139 |
| Respiratory | 65 (73.0) | 61 (80.3) |  |
| Endoscopy | 19 (21.3) | 8 (10.5) |  |
| Neurology | 5 (5.6) | 7 (9.2) |  |
| ARDS diagnosis |  |  |  |
| Time from ICU admission to ARDS onset, days | 2.0 (1.0–5.0) | 2.0 (1.0–4.0) | 0.332 |
| PaO_2_/FiO_2_ at ARDS onset | 108 (81–153) | 127 (98–172) | 0.088 |
| Worst PaO_2_/FiO_2_ during ICU stay | 84 (64–114) | 106 (79–124) | 0.020 |
| Risk factors for ARDS |  |  | 0.824 |
| Pneumonia | 64 (71.9) | 49 (64.5) |  |
| Extrapulmonary sepsis | 18 (20.2) | 20 (26.3) |  |
| Blood transfusion | 5 (5.6) | 4 (5.3) |  |
| Other risk factor | 1 (1.1) | 1 (1.3) |  |
| No risk factor | 1 (1.1) | 2 (2.6) |  |
| Organ support and ARDS management during ICU stay |  |  |  |
| Renal replacement therapy | 46 (51.7) | 48 (63.2) | 0.138 |
| Vasopressor | 86 (96.6) | 75 (98.7) | 0.625 |
| Neuromuscular blockers^†^ | 46 (51.7) | 51 (67.1) | 0.001 |
| Prone position^‡^ | 6 (6.7) | 27 (35.5) | <0.001 |
| Nitric oxide^‡^ | 19 (21.3) | 20 (26.3) | 0.002 |

Data are expressed as median (interquartile range) for quantitative variables and as *n*(%) for qualitative variables.

ICU, intensive care unit; ARDS, acute respiratory distress signal; PaO2/FiO2, partial pressure of arterial oxygen/fraction of inspired oxygen ratio

^*^Based on the worst PaO_2_/FiO_2_ ratio during ICU stay

^†^152 patient

^‡^151 patients

**Table S8. Acute respiratory distress syndrome characteristics and management, comparison between patients admitted to the intensive care unit for acute respiratory failure and those admitted for other reasons**

| **Characteristics** | **Admitted for acute respiratory failure**  **(n=86)** | **Admitted for other cause**  **(n=79)** | ***P*-value** |
| --- | --- | --- | --- |
| Initial respiratory management and intubation |  |  |  |
| Noninvasive ventilation | 22 (25.6) | 8 (10.1) | 0.018 |
| High-flow oxygen | 6 (7.0) | 6 (7.0) | 0.851 |
| Intubation criteria |  |  | <0.001 |
| Respiratory | 84 (97.7) | 42 (53.2) |  |
| Endoscopy | 1 (1.2) | 26 (32.9) |  |
| Neurology | 1 (1.2) | 11 (13.9) |  |
| ARDS diagnosis |  |  |  |
| Time from ICU admission to ARDS onset, days | 1.0 (0.0–3.0) | 3.0 (1.0–6.0) | <0.001 |
| PaO_2_/FiO_2_ at ARDS onset | 108 (80–156) | 128 (100–162) | 0.051 |
| Worst PaO_2_/FiO_2_ during ICU stay | 96 (69–118) | 98 (67–140) | 0.627 |
| Risk factors for ARDS |  |  | 0.042 |
| Pneumonia | 63 (73.3) | 45 (57.0) |  |
| Extrapulmonary sepsis and other risk factors | 23 (26.7) | 34 (43.0) |  |
| Organ support and ARDS management during ICU stay |  |  |  |
| Renal replacement therapy | 44 (51.2) | 50 (63.3) | 0.157 |
| Vasopressor | 84 (97.7) | 77 (97.5) | >0.999 |
| Neuromuscular blockers^†^ | 56 (70.1) | 41 (58.3) | 0.162 |
| Prone position^‡^ | 24 (29.7) | 9 (13.0) | 0.016 |
| Nitric oxide^‡^ | 24 (29.7) | 15 (21.2) | 0.269 |

Data are expressed as median (interquartile range) for quantitative variables and as *n*(%) for qualitative variables.

ICU, intensive care unit; ARDS, acute respiratory distress signal; PaO2/FiO2, partial pressure of arterial oxygen/fraction of inspired oxygen ratio

^*^Based on the worst PaO_2_/FiO_2_ ratio during ICU stay

^†^152 patient

^‡^151 patients

**Table S9. E-values for significant factors**

| **Variable** | **E-value (point estimate)** | **E-value (CI limit)** |
| --- | --- | --- |
| MELD score at ICU admission, *per 10-point increase* | 2.410 | 1.73 |
| PaO_2_/FiO_2_ ratio at ARDS diagnosis, *per 100-point increase* | 2.220 | 1.15 |
| Primary cause of ICU admission is acute respiratory failure | 2.390 | 1.13 |

E-values quantify the minimum strength of association that an unmeasured confounder would need with both the predictor and the outcome (on the risk-ratio scale), conditional on measured covariates, to fully explain away the observed association. For protective associations (OR<1), E-values are computed on the reciprocal. Confidence intervals (CI) limit E-values correspond to the confidence limit closest to the null.

CI, confidence intervals; MELD, model for end-stage liver disease; ICU, intensive care unit; PaO_2_/FiO_2_, partial pressure of arterial oxygen/fraction of inspired oxygen rate; ARDS, acute respiratory distress signal

**Table S10. Sensitivity analysis: Firth penalized logistic regression for 28-day mortality**

| **Variable** | **Adjusted OR (95% CI)** | **p-value** |
| --- | --- | --- |
| Age, *per year increase* | 1.01 (0.98–1.05) | 0.348 |
| Sex, *male* | 1.87 (0.78–4.92) | 0.169 |
| MELD score at ICU admission, *per 10-point increase* | 2.21 (1.43–3.58) | <0.001 |
| PaO_2_/FiO_2_ ratio at ARDS diagnosis, *per 100-point increase* | 0.50 (0.26–0.97) | 0.041 |
| Primary cause of ICU admission is acute respiratory failure | 0.46 (0.20–0.99) | 0.048 |

Odds ratios (OR) and 95% confidence intervals (CI) computed using univariate and multivariate logistic regression.

MELD, model for end-stage liver disease; ICU, intensive care unit; PaO_2_/FiO_2_, partial pressure of arterial oxygen/fraction of inspired oxygen rate; ARDS, acute respiratory distress signal

**Table S11. Sensitivity analysis: Logistic regression for 28-day mortality with ARDS onset within 48 hours of ICU admission**

| **Variable** | **Adjusted OR (95% CI)** | **p-value** |
| --- | --- | --- |
| Age, *per year increase* | 1.02 (0.99–1.06) | 0.218 |
| Sex, *male* | 1.80 (0.73–4.82) | 0.216 |
| MELD score at ICU admission, *per 10-point increase* | 2.54 (1.61–4.23) | <0.001 |
| PaO_2_/FiO_2_ ratio at ARDS diagnosis, *per 100-point increase* | 0.44 (0.21–0.90) | 0.026 |
| ARDS onset within 48 hours of ICU admission | 0.40 (0.16–0.90) | 0.031 |

Odds ratios (OR) and 95% confidence intervals (CI) computed using univariate and multivariate logistic regression.

MELD, model for end-stage liver disease; ICU, intensive care unit; PaO_2_/FiO_2_, partial pressure of arterial oxygen/fraction of inspired oxygen rate; ARDS, acute respiratory distress signal

**Table S12. Sensitivity analysis: Logistic regression for 28-day mortality with ARDS onset within 72 hours of ICU admission**

| **Variable** | **Adjusted OR (95% CI)** | **p-value** |
| --- | --- | --- |
| Age, *per year increase* | 1.02 (0.99–1.06) | 0.185 |
| Sex, *male* | 1.59 (0.64–4.23) | 0.330 |
| MELD score at ICU admission, *per 10-point increase* | 2.47 (1.58–4.09) | <0.001 |
| PaO_2_/FiO_2_ ratio at ARDS diagnosis, *per 100-point increase* | 0.44 (0.21–0.90) | 0.025 |
| ARDS onset within 72 hours of ICU admission | 0.35 (0.13–0.88) | 0.033 |

Odds ratios (OR) and 95% confidence intervals (CI) computed using univariate and multivariate logistic regression.

MELD, model for end-stage liver disease; ICU, intensive care unit; PaO_2_/FiO_2_, partial pressure of arterial oxygen/fraction of inspired oxygen rate; ARDS, acute respiratory distress signal

**Table S13. Sensitivity analysis: Logistic regression for 28-day mortality adding period of admission 2007–2014**

| **Variable** | **Adjusted OR (95% CI)** | **p-value** |
| --- | --- | --- |
| Age, *per year increase* | 1.02 (0.98–1.05) | 0.336 |
| Sex, *male* | 2.15 (0.86–5.96) | 0.117 |
| MELD score at ICU admission, *per 10-point increase* | 2.27 (1.44–3.75) | 0.001 |
| PaO_2_/FiO_2_ ratio at ARDS diagnosis, *per 100-point increase* | 0.52 (0.26–1.05) | 0.066 |
| ICU admission for acute respiratory failure | 0.45 (0.19–1.01) | 0.058 |
| Period of admission 2007–2014 | 1.69 (0.77–3.78) | 0.192 |

Odds ratios (OR) and 95% confidence intervals (CI) computed using univariate and multivariate logistic regression.

MELD, model for end-stage liver disease; ICU, intensive care unit; PaO_2_/FiO_2_, partial pressure of arterial oxygen/fraction of inspired oxygen rate; ARDS, acute respiratory distress signal

**Table S14. Distribution of identified pathogens in documented infections: descriptive analysis.**

|  | **Documented infection in all patients**  **n=87** | **Documented infection among patients with pneumonia as ARDS risk factor**  **n=65** | **Documented infection among patients with extra-pulmonary sepsis as ARDS risk factor**  **n=22** |
| --- | --- | --- | --- |
| Gram-negative bacilli | 43 (49.4) | 32 (49.2) | 11 (50.0) |
| *Pseudomonas aeruginosa* | 15 (17.2) | 13 (20.0) | 2 (9.1) |
| Other | 28 (32.2) | 19 (29.2) | 9 (40.9) |
|  |  |  |  |
| Gram-positive cocci | 19 (21.8) | 11 (16.9) | 8 (36.4) |
| *Staphylococcus aureus* | 6 (6.9) | 5 (7.7) | 1 (4.5) |
| *Enterococcus* | 9 (10.3) | 2 (3.1) | 7 (31.8) |
| *Streptococcus pneumoniae* | 4 (4.6) | 4 (6.2) | 0 (0.0) |
|  |  |  |  |
| Fungi | 16 (18.4) | 13 (20.0) | 3 (13.6) |
| *Candida albicans* | 5 (5.7) | 2 (3.1) | 3 (13.6) |
| *Aspergillosis fumigatus* | 2 (2.3) | 2 (3.1) | 0 (0.0) |
| *Pneumocystis jirovecii* | 9 (10.3) | 9 (13.8) | 0 (0.0) |
|  |  |  |  |
| Viral | 8 (9.2) | 8 (12.3) | 0 (0.0) |
| *Influenza* | 1 (1.1) | 1 (1.5) | 0 (0.0) |
| SARS-CoV-2 | 5 (5.7) | 5 (7.7) | 0 (0.0) |
| Cytomegalovirus | 2 (2.3) | 2 (3.1) | 0 (0.0) |
|  |  |  |  |
| *Mycobacterium tuberculosis* | 1 (1.1) | 1 (1.5) | 0 (0.0) |

Qualitative variables are expressed as number (percentage).

ARDS, acute respiratory distress signal; SARS-CoV-2, severe acute respiratory syndrome coronavirus-2

**Figure S1: Calibration of the multivariable logistic model at day 28**


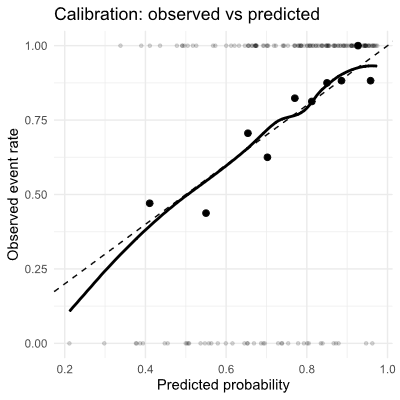


Observed event rate versus predicted probability. The dashed 45° line indicates perfect calibration; the solid curve is a loess smooth of observed vs predicted; black dots are decile-binned observed rates; light gray ticks show individual predictions/outcomes. The model shows overall good calibration with only minor deviations at the extremes.
